# SmartArc-based volumetric modulated arc therapy for endometrial cancer: a dosimetric comparison with helical tomotherapy and intensity-modulated radiation therapy

**DOI:** 10.1186/1471-2407-13-515

**Published:** 2013-11-01

**Authors:** Ruijie Yang, Junjie Wang, Shouping Xu, Hua Li

**Affiliations:** 1 Department of Radiation Oncology, Peking University Third Hospital, Beijing, China; 2 Department of Radiation Oncology, General Hospital of the People’s Liberation Army, Beijing, China; 3 Department of Obstetrics & Gynecology, Peking University Third Hospital, Beijing, China

**Keywords:** Endometrial cancer, Helical tomotherapy, Intensity-modulated radiation therapy, Volumetric modulated arc therapy

## Abstract

**Background:**

The purpose of the present study was to investigate the feasibility of using volumetric modulated arc therapy with SmartArc (VMAT-S) to achieve radiation delivery efficiency higher than that of intensity-modulated radiotherapy (IMRT) and helical tomotherapy (HT) when treating endometrial cancer, while maintaining plan quality.

**Methods:**

Nine patients with endometrial cancer were retrospectively studied. Three plans per patient were generated for VMAT-S, IMRT and HT. The dose distributions for the planning target volume (PTV), organs at risk (OARs) and normal tissue were compared. The monitor units (MUs) and treatment delivery time were also evaluated.

**Results:**

The average homogeneity index was 1.06, 1.10 and 1.07 for the VMAT-S, IMRT and HT plans, respectively. The V_40_ for the rectum, bladder and pelvis bone decreased by 9.0%, 3.0% and 3.0%, respectively, in the VMAT-S plan relative to the IMRT plan. The target coverage and sparing of OARs were comparable between the VMAT-S and HT plans. The average MU was 823, 1105 and 8403 for VMAT-S, IMRT and HT, respectively; the average delivery time was 2.6, 8.6 and 9.5 minutes, respectively.

**Conclusions:**

For endometrial cancer, the VMAT-S plan provided comparable quality with significantly shorter delivery time and fewer MUs than with the IMRT and HT plans. In addition, more homogeneous PTV coverage and superior sparing of OARs in the medium to high dose region were observed in the VMAT-S relative to the IMRT plan.

## Background

Endometrial cancer is one of the most common gynecologic cancers in the world. Whole pelvic radiation therapy (WPRT) can reduce the rate of pelvic disease recurrence in patients who have undergone hysterectomy for endometrial cancer [1,2]. For whole pelvic radiation therapy, intensity-modulated radiation therapy (IMRT) and helical tomotherapy (HT) have been shown to give a more conformal dose distribution than conventional radiotherapy, with better sparing of adjacent critical structures [3-6]. However, the IMRT and HT techniques also have drawbacks. The prolonged treatment delivery time required for IMRT and HT relative to three-dimensional conformal radiotherapy may worsen the accuracy of treatment because of increased intra-fractional patient motion. Additionally, patient throughput is reduced using IMRT and HT with economic consequences. Another issue of concern is the higher number of monitor units (MU) used in IMRT and HT, which can increase the number of secondary cancers after curative treatment [7]. Recently, volumetric modulated arc therapy (VMAT) has been introduced to address the above mentioned issues. The potential benefits involved in the use of VMAT relative to standard IMRT are obtained with enhanced degrees of freedom in continuously modulating the multileaf collimator (MLC) field shape, gantry rotation speed and dose rate. However, the potential advantages of VMAT are highly dependent on the actual optimization algorithm in the treatment planning system (TPS). Only algorithms which handle the increased degrees of freedom appropriately will have the potential to achieve the potential advantages offered by VMAT. It is therefore important to validate the clinical applicability of VMAT algorithms. The performance of RapidArc (the VMAT algorithm used in Eclipse TPS plans for Varian accelerators) has been shown to provide superior or equivalent dose distributions relative to standard IMRT for the treatment of prostate, cervical, anal canal, lung, brain and head and neck cancer within the preliminary planning studies [8-13].

Recently, the VMAT optimizer in the Pinnacle3 SmartArc treatment planning module (Philips Medical Systems, Madison, WI, USA) was used in combination with a Varian Trilogy linear accelerator in our department. Studies regarding the clinical performance of these systems are therefore of interest. In addition, more radiation fields are used in VMAT and HT than in IMRT. Consequently, a greater volume of normal tissue will be exposed to lower radiation doses. There are some concerns with regard to the increase in the normal tissue (NT) integral dose using VMAT as a potential risk factor for the development of secondary cancers. For a better assessment of the risks of the development of a second malignancy, it is necessary to evaluate the integral dose (ID) deposited in critical structures and normal tissue. To date, no study has been published concerning the evaluation of the dosimetry for WPRT using SamrtArc-based VMAT (VMAT-S) and the Varian linear accelerator in the treatment of postoperative endometrial cancer patients, especially in terms of the ID to NT and organs at risk (OARs). The aim of the present study was to compare the VMAT-S plan with the IMRT and HT plans for whole pelvic radiation therapy involving postoperative endometrial cancer patients, with a focus on the volume of NT and OARs receiving low radiation doses, and the IDs deposited in NT and OARs.

## Methods

### Patient selection and simulation

Nine consecutive patients who had been treated with postoperative WPRT for endometrial cancer were retrospectively selected for this study. The study was approved by Ethics Committee of Peking University Third Hospital and informed consent was obtained. All patients had undergone total abdominal hysterectomy and bilateral salpingo-oophorectomy, pelvic and/or para-aortic lymph node dissection/sampling, with no gross residual disease. Of the 9 patients, 7 were simulated and treated in the supine position and 2 in the prone position on a belly board. A vaginal marker was carefully inserted to indicate the position of the vaginal apex, without distortion of the vagina. All patients were instructed to drink 1500 ml of water at 1 hour before simulation and treatment; they were immobilized using a thermoplastic mask and scanned from the T12 vertebrate to mid-thigh using oral and i.v. contrast. The image sets were transferred to the Pinnacle planning system for contouring and planning.

### Definition and contour of targets

The clinical target volume (CTV) was delineated according to the consensus guidelines of the RTOG, GOG, NCIC, ESTRO and ACR groups [14]. The CTV included pelvic lymph node regions (common, internal and external iliacs), the proximal 3.0 cm of the vagina and paravaginal tissues for all of the patients. For patients with cervical stromal invasion, the presacral lymph node region was also contoured to the inferior border of the S2 vertebra. A margin of 0.7 cm was added to the “vessels” contour in all dimensions and modified using anatomic boundaries (as clinically indicated for individual patients) to create the nodal clinical target volume, from which the pelvic bones, femoral heads and vertebral bodies were excluded. The CTV was expanded by 1 cm to create the planning target volume (PTV).

### Definition and contour of OARs and NT

Contours for OARs included the bladder, rectum, small intestine, colon and pelvic bones. The superior and inferior extents of OARs were outlined on all CT slices in which portions of the PTV existed, as well as at an additional 2 cm superior and inferior to the limits of the PTV. The rectum was defined from the rectosigmoid flexure to the anus. The small intestine and colon were contoured together as one structure referred to as the “bowel”. The bowel volume was contoured as individual loops. The pelvic bones were defined and contoured according to a previously published study [15]. The external contours of all the bones within the pelvis were delineated for each patient. The entire bony contour was divided into three subsites: the ilium, lower pelvis and lumbosacral spine. No expansion of any of these OARs was made to account for organ motion and set up error. The whole body was contoured as the entire volume of all slices where the PTV existed, as well as at an additional 2 cm superior and inferior to the PTV. The NT was defined as the whole body within the skin surface minus the PTV.

### Treatment planning

The VMAT-S, IMRT and HT plans were all generated using 6-MV photon beams for each patient. The VMAT-S and IMRT plans were created using a Philips Pinnacle planning system, version 9.2 (Philips Radiation Oncology Systems, Fitchburg, WI, USA), for delivery using a Varian Trilogy linear accelerator equipped with a Millennium MLC. The HT plan was generated using a tomotherapy planning system (Hi-Art Tomotherapy 2.2.4.1, TomoTherapy, Madison, WI, USA). All plans were generated for VMAT-S, IMRT and HT using the same plan objectives (Table 1).

**Table 1:** The dose-volume objectives and constraints used in VMAT-S, IMRT and HT plans

**Structures**	**Objectives and constraints**
PTV	Minimal dose, 47.5 Gy; maximal dose, 55 Gy; ≥95% of PTV receiving 50 Gy
Bowel	≤35% of bowel receiving ≥35 Gy
Bladder	≤40% of bladder receiving ≥40 Gy
Rectum	≤60% of rectum receiving ≥40 Gy

IMRT plan optimization was performed using the Direct Machine Parameter Optimization algorithm in the Pinnacle3 treatment planning system. Based on the findings of previous studies [5,16], nine coplanar beams were used. Fields were set with an equal spacing of 40° and a starting angle of 0°. The minimum segment area was set to 5 cm^2^ and the minimum number of segment MUs was five. A collapsed-cone convolution algorithm was used to calculate the dose distribution, with a dose grid resolution of 4 mm.

The VMAT-S plans were optimized using the Pinnacle3 SmartArc module. The details regarding the SmartArc planning algorithm have been described by Bzdusek et al. [17]. All VMAT-S plans were generated using one dual arc, the first clockwise from 181–179°, and the second counterclockwise from 179–181°, with a final control point resolution of 2°. To allow maximal modulation per arc, no limitation on the delivery time was used during the optimization. Continuous gantry motion, dose-rate variation and MLC motion were approximated by optimizing individual beams at 2° gantry angle increments. The choice of this resolution was based on preliminary planning exercises to get better plan quality utilizing the higher degree of modulation. Other planning parameters were MLC motion speed 0–2.5 cm/s, gantry rotation speed 0.5–4.8 degrees/s and dose rate 0–600 MU/min.

For HT plans, CT datasets with structures that had been contoured in the Pinnacle system were transferred to the Tomotherapy planning system using the Digital Imaging and Communication in Medicine RT protocol. The optimization was guided using dose volume objectives and constraints, precedence, importance and penalty parameters, which were set based on the results of IMRT and our pilot study. The field width was 2.5 cm, the pitch (ratio of the distance traveled by the treatment couch per rotation to the fan beam thickness) was 0.3 and the modulation factor was 3.0.

### Dosimetric comparison

For the convenience of comparison, all plans were normalized to deliver 50 Gy to 95% of the PTV in 25 fractions. The DVHs of the VMAT-S, IMRT and HT plans were compared in terms of coverage of the PTV, OARs and normal tissue sparing, and the ID deposited in the OARs and NT. The parameters analyzed included the percentage of the PTV that received 95%, 100%, 105% and 110% of the prescription dose (PTV_95_, PTV_100_, PTV_105_ and PTV_110_, respectively), the homogeneity index (HI) and the conformity index (CI). The HI was defined as the minimum dose in 5% of the PTV/minimum dose in 95% of the PTV (D5%/D95%). The lower (closer to 1) the HI is, the better the dose homogeneity. Since not all regions of the PTV were covered by the prescribed dose, the CI was calculated as follows: CI = CF (cover factor) × SF (spill factor), where the CF was defined as the percentage of the PTV volume receiving at least the prescribed dose and the SF as the volume of the PTV receiving at least the prescription dose relative to the total prescription dose volume. The closer the CI value is to 1, the better the dose conformity. To quantify the dose distribution of OARs and NT at different dose levels, the percentage volume of the OARs and NT receiving a dose of 10, 20, 30, 40 and 50 Gy (V_10_, V_20_, V_30_, V_40_ and V_50_, respectively) were evaluated and compared in the VMAT-S, IMRT and HT plans. The mean dose and ID deposited in the OARs and NT were also compared. The ID is equal to the mean dose multiplied by the volume of each structure.

### Statistics

Dosimetric differences regarding VMAT-S were compared with those regarding IMRT and HT. Statistical significance was evaluated using the paired two-tailed Student t test. A 2-tailed *P*-value < 0.05 was considered as being statistically significant. Analyses were performed using the Statistical Package for Social Science, version 13.0, software (SPSS, Chicago, IL, USA).

## Results

### PTV coverage

**Figure 1:**
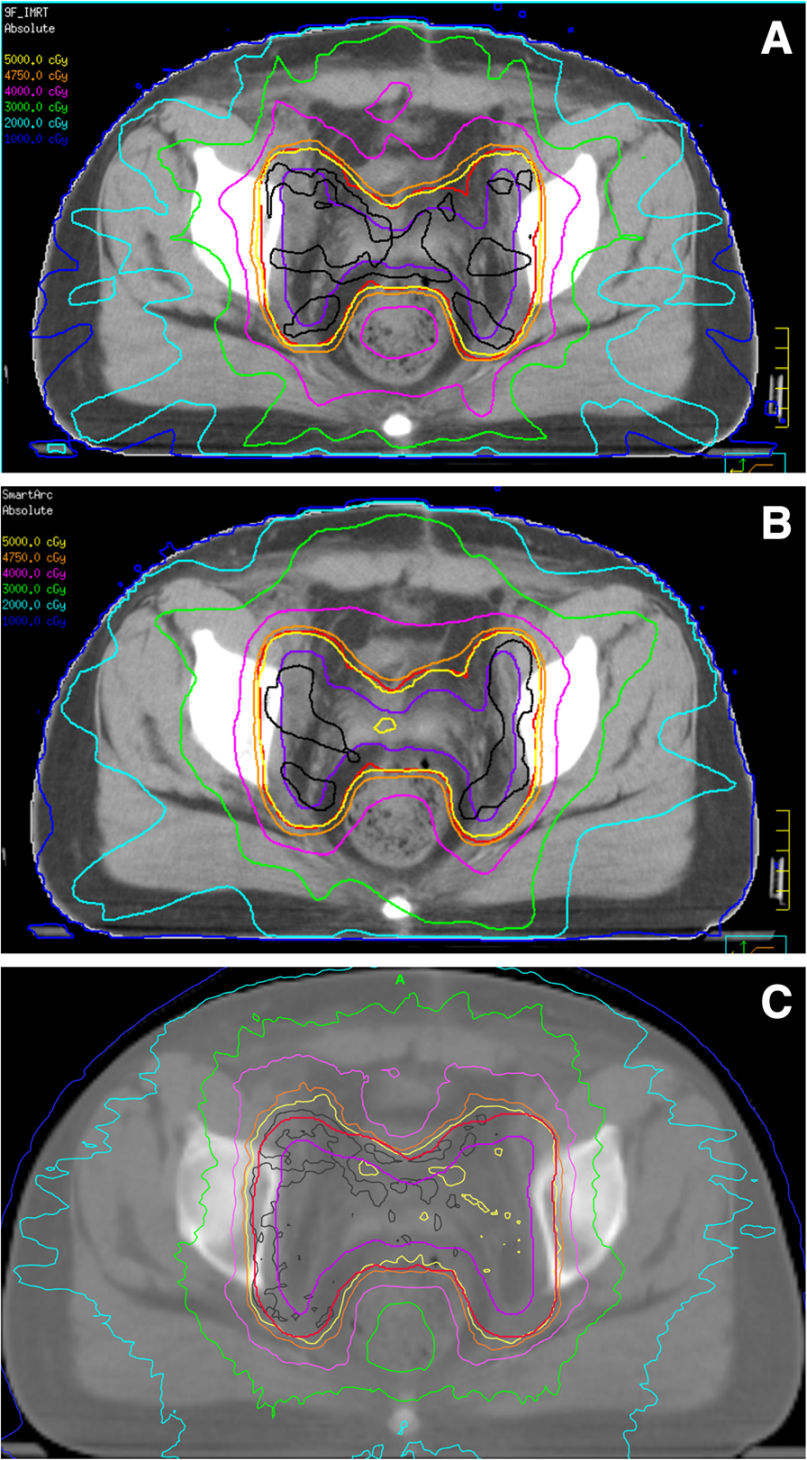
**Representative axial computed tomography slices showing isodose distributions. (A)** IMRT. **(B)** VMAT-S. **(C)** Tomotherapy. PTV is shown in red, CTV in slate blue. Isodose lines are indicated as follows: inverse grey, 5250 cGy; yellow, 5000 cGy; orange, 4750 cGy; purple, 4000 cGy; green, 3000 cGy; sky blue, 2000 cGy; and blue 1000 cGy.

**Figure 2:**
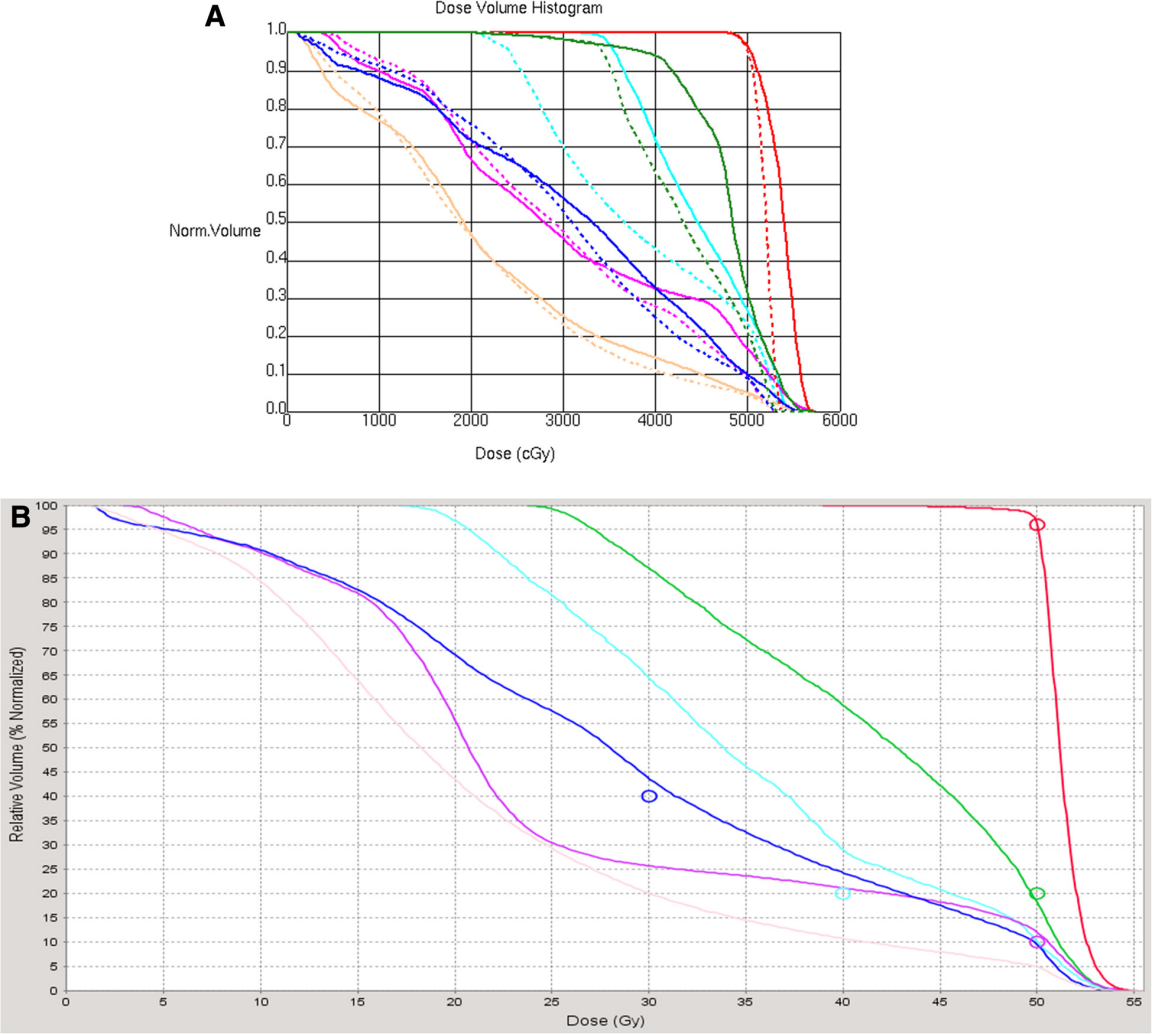
**Representative dose–volume histograms for (a) IMRT Vs VMAT-S, (b) tomotherapy.** The curves of IMRT and Tomotherapy indicated in solid line, those of SmartArc indicated in dashed lines. The colors of the curves indicated as follows: red, PTV; forest, rectum; skyblue, bladder; purple, bowel; blue, pelvic bones; skin, normal tissue.

For all 9 cases, clinically acceptable plans could be generated for VMAT-S, IMRT and HT. The typical dose distribution and the dose volume histogram comparison were given in Figures 1 and 2. The data for PTV coverage are summarized in Table 2. The VMAT-S plan significantly improved the PTV dose homogeneity as compared with the IMRT plan. No significant difference was found in PTV dose homogeneity between the VMAT-S and HT plans. The average HI was 1.06, 1.10 and 1.07 for the VMAT-S, IMRT and HT plans, respectively. The mean conformity index was 0.89, 0.87 and 0.87 for the VMAT-S, IMRT and HT plans, respectively; the difference in conformity between the VMAT and IMRT or HT plans was not statistically significant. Specifically, for the VMAT-S, IMRT and HT plans the mean PTV_105_ was 40.5%, 67.1% and 16.7%, respectively, and the mean PTV_110_ was 0.00%, 5.30% and 0.20%, respectively. The average PTV_100_ was 95.1%, 95.6% and 95.8% for the VMAT-S, IMRT and HT plans, respectively. No difference in PTV D_mean_ and ID between the VMAT-S and IMRT, or HT plans was found.

**Table 2:** Summary of PTV coverage data for VMAT-S, IMRT and HT plans $\left(\bar{x}\pm \sigma \right)$

	**PTV**_**95 **_**(%)**	**PTV**_**100 **_**(%)**	**PTV**_**105 **_**(%)**	**PTV**_**110 **_**(%)**	**D**_**mean **_**(Gy)**	**CI**	**HI**
**Mean (VMAT-S)**	99.82	95.10	40.50	0.00	52.01	0.89	1.06
**Mean (IMRT)**	99.91	95.60	67.10	5.30	52.51	0.87	1.10
***P***	0.53	0.68	0.03	0.01	0.51	0.07	0.01
**Mean (HT)**	99.61	95.80	16.70	0.20	51.45	0.87	1.07
***P***	0.37	0.83	0.00	0.71	0.08	0.06	0.25

PTV: planning target volume, VMAT-S: volumetric modulated arc therapy with SmartArc, IMRT: intensity-modulated radiotherapy, HT: helical tomotherapy, D_mean_: mean dose, CI: conformity index, HI: homogeneity index.

### OARs and NT sparing

The dose-volume histogram data for the OARs and NT are listed in Table 3. As compared with the IMRT plan, the VMAT-S plan significantly reduced the irradiated volume of the OARs and NT receiving medium to high doses. For the rectum, the V_30_ and V_40_ decreased by 11.0% and 9.0%, respectively. The V_30_ and V_40_ of pelvis bone decreased by 5.0% and 3.0%, respectively. The V_30_ and V_40_ of the bladder also decreased by 3.0% and 3.0%, respectively. However the VMAT-S plan slightly increased the volume of the bowel, bladder and pelvis bone receiving doses <20 Gy relative to the IMRT plan. The V_20_ increased by 4.0%, 5.0% and 8.0% for the bowel, bladder and pelvis bone, respectively. In addition, the V_5_, V_10_ and V_20_ of the NT also increased by 6.0%, 11.0% and 3.0%, respectively. When comparing the VMAT-S plans with the HT plans, the sparing of the OARs and NT was found to be very similar. Even the volumes receiving more than 20 Gy in the OARs were reduced using the HT plan, while the low dose volumes of the OARs were increased, but the difference was not statistically significant.

**Table 3:** Summary of OARs and NT dose distribution for VMAT-S, IMRT and HT plans $\left(\bar{x}\pm \sigma \right)$

**Structures**		**V**_**10**_	**V**_**20**_	**V**_**30**_	**V**_**40**_	**V**_**50**_	**D**_**mean **_**(Gy)**
Bowel	Mean (VMAT-S)	0.88	0.70	0.39	0.22	0.08	27.35
	Mean (IMRT)	0.83	0.66	0.40	0.22	0.09	26.72
	*P*	0.02	0.03	0.15	0.27	0.39	0.02
	Mean (HT)	0.91	0.72	0.38	0.19	0.08	26.91
	*P*	0.07	0.08	0.13	0.10	0.61	0.03
Rectum	Mean (VMAT-S)	0.99	0.96	0.82	0.54	0.25	40.33
	Mean (IMRT)	0.98	0.96	0.93	0.63	0.27	42.40
	*P*	0.19	0.52	0.03	0.00	0.06	0.01
	Mean (HT)	1.00	0.98	0.85	0.57	0.25	40.99
	*P*	0.21	0.31	0.31	0.08	0.36	0.12
Bladder	Mean (VMAT-S)	1.00	0.96	0.67	0.35	0.16	36.23
	Mean (IMRT)	0.99	0.91	0.70	0.38	0.17	36.33
	*P*	0.36	0.03	0.03	0.04	0.37	0.17
	Mean (HT)	1.00	0.86	0.65	0.33	0.14	35.09
	*P*	0.52	0.02	0.16	0.08	0.38	0.06
Pelvic bones	Mean (VMAT-S)	0.91	0.73	0.40	0.22	0.08	27.85
	Mean (IMRT)	0.84	0.65	0.45	0.25	0.09	27.28
	*P*	0.00	0.00	0.03	0.03	0.17	0.13
	Mean (HT)	0.91	0.69	0.41	0.24	0.09	28.22
	*P*	0.51	0.08	0.07	0.06	0.53	0.61
Normal tissue	Mean (VMAT-S)	0.79	0.45	0.21	0.11	0.05	20.48
	Mean (IMRT)	0.68	0.42	0.21	0.12	0.05	19.77
	*P*	0.00	0.03	0.15	0.15	0.25	0.08
	Mean (HT)	0.81	0.42	0.17	0.06	0.03	20.56
	*P*	0.35	0.10	0.07	0.09	0.12	0.15

OARs: organs at risk, NT: normal tissue, VMAT-S: volumetric modulated arc therapy with SmartArc, IMRT: intensity-modulated radiotherapy, HT: helical tomotherapy, V_10_, V_20_, V_30_, V_40_ and V_50_: the percentage volume of the OARs and NT receiving a dose of 10, 20, 30, 40 and 50 Gy, respectively, D _mean_: mean dose.

### Integral dose to the OARs and NT

The integral doses deposited in the OARs and NT using the VMAT-S, IMRT and HT plans are given in Table 4. No significant difference was found using the VMAT-S plans relative to the IMRT or HT plans.

**Table 4:** ID delivered to the OARs and NT for the VMAT-S, IMRT and HT plans $\left(\bar{x}\pm \sigma \right)$

**Plans**	**VMAT-S**	**IMRT**		**HT**	
	**Mean**	**Mean**	***P***	**Mean**	***P***
	**(Gy × L)**	**(Gy × L)**		**(Gy × L)**	
**Bowel**	23.83	23.22	0.35	23.45	0.21
**Rectum**	3.18	3.34	0.36	3.26	0.32
**Bladder**	11.40	11.98	0.17	11.02	0.20
**Pelvic bones**	36.60	36.10	0.21	37.50	0.35
**Normal tissue**	262.30	258.50	0.11	265.12	0.09

ID: integral dose, OARs: organs at risk, NT: normal tissue, VMAT-S: volumetric modulated arc therapy with SmartArc, IMRT: intensity-modulated radiotherapy, HT: helical tomotherapy.

### MU and treatment delivery time

The MU was on average 1105 for IMRT, 823 for VMAT-S and 8403 for HT. As compared with IMRT, the MU was reduced by 25.5% using VMAT. The treatment delivery time was on average 8.6 minutes for IMRT, 2.6 minutes for VMAT-S and 9.5 minutes for HT. Relative to IMRT and HT, the average treatment time was reduced by 6.0 minutes (69.8%) and 6.9 minutes (72.6%), respectively using the VMAT plan. The treatment delivery time was defined as the time from first beam turn on until last beam turn off.

## Discussion

We evaluated the VMAT plans based on SmartArc using a Varian Trilogy linear accelerator; this accelerator is now used clinically for the treatment of endometrial cancer in our department, a complex situation often encountered in the clinic. As compared with the IMRT plan, the VMAT-S plan provided a more homogeneous dose distribution in the PTV and better sparing of the OARs and NT in the medium to high dose region; a slightly larger volume of normal tissue received a radiation dose of 20 Gy. No significant difference was found between the VMAT-S and HT plans. The major benefits of VMAT-S plan were manifested in the faster delivery time and lower MU relative to the IMRT and HT plans. Luca et al. [18] compared fixed field IMRT with VMAT for cervical cancer as planned/delivered using an Eclipse/Varian linear accelerator. They also found that RapidArc improved dose homogeneity and sparing of the rectum, bladder and small bowel in the medium to high dose region.

The volumes receiving doses of >30 Gy in the bladder, rectum and pelvis bone were reduced using the VMAT plan relative to the IMRT plan, whereas the volumes receiving doses <20 Gy were increased for the bladder and pelvis bone. This was because in IMRT the dose is delivered using relatively few beam angles as compared with VMAT. The improved sparing of the bladder, rectum and pelvis bone at medium to high doses using VMAT as compared with IMRT is expected to further reduce the acute and late toxicities, especially for patients requiring a local boost and concurrent/sequential chemotherapy. This is also relevant to patients not suitable for the high dose rate boost. As an arc-based approach to the delivery of IMRT, VMAT can deliver a more homogeneous dose to the target volume with a greater degree of freedom of intensity modulation. As expected, greater volumes of bowel, pelvic bones and NT received radiation doses ranging from 5–20 Gy, as compared with IMRT. The increased low dose bath effect in the NT and pelvic bones might be explained by the larger and longer target volumes exposed to more radiation beams in the arc pattern of radiation delivery involved in VMAT. Lian et al. [6] also found that in postoperative endometrial cancer patients the use of HT increased low dose irradiation of the normal tissue and skeleton in pelvic and para-aortic radiotherapy. A greater volume of pelvic bones exposed to a dose of 2–20 Gy could increase the risk of hematologic suppression [14,15] and bone fracture [19]. A larger volume of NT received a low dose of 2–20 Gy using VMAT-S relative to IMRT. Some concerns have been raised regarding the risk of secondary cancers in NT irradiated to low dose. Given the better sparing of OARs, and the longer life expectancy of older patients with endometrial cancer treated using VMAT-S, its benefits outweigh its pitfalls. Investigation of this issue was beyond the scope of this study, and has previously been addressed and discussed [7]. It is possible that the low dose volume in the pelvic bones and NT could be decreased in the planning process by introducing the corresponding dose volume constraints in VMAT-S and HT. Because the present study was designed to be a comparative dosimetric evaluation of VMAT-S, IMRT and HT plans, we did not use any constraints regarding the pelvic bones and NT, and used the same dose volume objectives and constraints in all three techniques based on our experience and a pilot study. Of course, it is possible that there may be slight differences in the results caused by the different optimization algorithms used in each of the unique planning systems.

VMAT-S and HT provided very similar and highly conformal plans. HT provided a more homogeneous dose distribution in the PTV105 (16.7% vs. 40.5%; *P* = 0.00), but no significant difference in terms of the HI (1.06 vs. 1.07; *P* = 0.25). The integral dose delivered to normal tissue was also equivalent using VMAT-S and HT in our study. Delivery of a statistically significant higher integral dose to normal tissue for has been reported for HT relative to VMAT in previous studies [20,21]. However, the difference was small (approximately 3%). The clinical relevance is very difficult to assess. A study published by D’Souza and Rosen [22] suggested that the total energy deposited in a patient is relatively independent of treatment planning parameters (such as beam orientation or relative weighting when many beams are used) for deep-seated targets. In addition, because bladder, rectum and bowel, and pelvis bone overlapped with the PTV, their maximum doses were correlated to the minimum dose delivered to the PTV. In the current study, the V_50_ for bladder, rectum, bowel and pelvis bone were all equivalent among three techniques.

The major benefits of VMAT-S were manifested in the faster treatment delivery time and lower MU as compared with IMRT and HT. The delivery time for IMRT is significantly higher than that for VMAT due to the multiple field arrangement, time to reposition the gantry and mode up signal of the Clinac for every field. The average treatment delivery time was reduced by more than 6 minutes using the VMAT plans as compared with IMRT and HT plans. This reduction in treatment delivery time is clinically relevant in relation to patient comfort and infra-fraction motion. Faster delivery could improve patient adherence to treatment and reduce intra-fractional motion. In addition, the higher delivery efficiency also allowed for more time to carry out image-guided radiotherapy, further reducing the treatment margin and toxicity. More patients could be treated per day using VMAT due to the short delivery time. In addition, the delivery efficiency of the SmartArc plans is higher in terms of requiring less MUs.

## Conclusions

In postoperative WPRT for endometrial cancer, VMAT-S provided more homogeneous PTV coverage and superior sparing of OARs in high radiation dose regions than IMRT, without significantly increasing the integral dose delivered to OARs; however, a greater volume of normal tissue was found to receive doses of <20 Gy. VMAT-S significantly improved treatment efficiency in terms of delivery time and MU relative to IMRT. As compared with HT, VMAT was able to provide an approximate 25% reduction in MU and a 7 minute reduction in treatment time while maintaining comparable plan quality. The clinical significance of these differences with regard to dosimetry and radiation delivery efficiency needs to be further investigated.

## Abbreviations

VMAT-S: Volumetric modulated arc therapy with SmartArc; IMRT: Intensity-modulated radiotherapy; HT: Helical tomotherapy; PTV: Planning target volume; CTV: Clinical target volume; OARs: Organs at risk; NT: Normal tissue; MU: Monitor unit; WPRT: Whole pelvic radiation therapy; MLC: Multileaf collimator; TPS: Treatment planning system; ID: Integral dose; CI: Conformity index; HI: Heterogeneity index.

## Competing interests

Ruijie Yang was funded by a grant project: National Natural Science Foundation of China (No. 81071237). Junjie Wang, Shouping Xu and Hua Li declare that they have no competing interests.

## Authors’ contributions

YR, WJ, XS and LH were responsible for the concept and design of the study. YR, XS and LH undertook data acquisition. Analysis and interpretation of data was carried out by YR, WJ and XS. YR and LH drafted the manuscript. All of the authors read and approved the final version of the manuscript.

## Pre-publication history

The pre-publication history for this paper can be accessed here:

http://www.biomedcentral.com/1471-2407/13/515/prepub
